# Correction: Lin, K.-H., et al. Molecular Functions of Thyroid Hormone Signaling in Regulation of Cancer Progression and Anti-Apoptosis. *Int. J. Mol. Sci.,* 2019, *20*, 4986

**DOI:** 10.3390/ijms21093185

**Published:** 2020-04-30

**Authors:** Yu-Chin Liu, Chau-Ting Yeh, Kwang-Huei Lin

**Affiliations:** 1 Department of Biochemistry, College of Medicine, Chang-Gung University, Taoyuan 333, Taiwan; k1506820@gmail.com; 2 Department of Biomedical Sciences, College of Medicine, Chang-Gung University, Taoyuan 333, Taiwan; 3 Liver Research Center, Chang Gung Memorial Hospital, Taoyuan 333, Taiwan; chauting@adm.cgmh.org.tw; 4 Research Center for Chinese Herbal Medicine, College of Human Ecology, Chang Gung University of Science and Technology, Taoyuan 333, Taiwan

The authors wish to make the following corrections to this paper [1]:

There were labeling mistakes in the column TRα2 displaying the T3 binding ability in the original version of Figure 1 (page 3). Circulating THs interact with thyroid hormone receptors to promote downstream signaling pathways and activate transcription factors. The four major TR isoforms, TRα1, TRα2, TRβ1, and TRβ2, are produced by *c-erbA*α and *c-erbAβ* genes. Their human homologs are designated THRA and THRB. The *c-erbA*α gene located on chromosome 17 encodes two different TRα isoforms. One is functional TH-binding TRα1 and the other is a dominant-negative splice variant, TR𝛼2, lacking TH binding activity [2]. T3 interacts with thyroid hormone receptors via C-terminal activation function-2 (AF-2) in the ligand-binding domain (LBD), however, only TRα2 has a distinct C-terminal extension and absent activation function-2 (AF-2) region, which suggested that TRα2 does not bind T3 [3]. TR𝛼2 is unique in regard to its lack of binding to THs while interacting with DNA, and its precise function is unclear at present. We have made a correction to show that the TRα2 did not bind T3 and marked presence or lack of presence of the AF-2 domain in Figure 1 as follows:

The authors would like to apologize for any inconvenience caused to the readers by these changes.

## Figures and Tables

**Figure 1:**
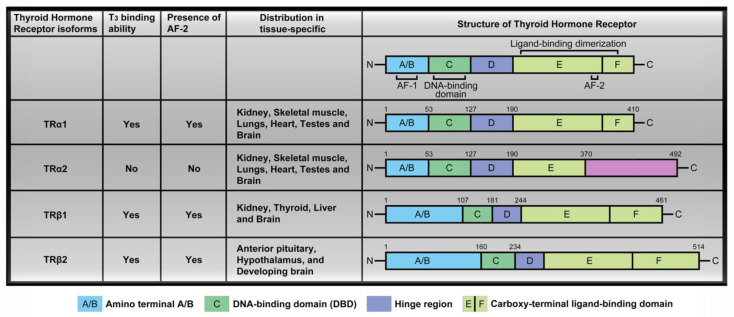
TR isoforms and structure distribution. Thyroid hormone receptors (TR) contain several domains, specifically, the amino terminal A/B that may function as a gene enhancer, the DNA-binding domain (DBD), the hinge region containing the nuclear localization signal and the carboxy-terminal ligand-binding domain that binds T_3_. The four major TR isoforms are TRα1, TRα2, TRβ1, and TRβ2. TH binding is widely distributed in a tissue-specific manner such as TRα1 and TRα2 expressed in the kidney, skeletal muscle, lungs, heart, and testes, with particularly high levels detected in the brain. TRβ1 expression is significant in the brain, thyroid, liver, and kidney while the TRβ2 isoform is specifically expressed in the anterior pituitary, hypothalamus, and developing brain.
